# The new ICD-11 diagnosis of personality disorder in forensic psychiatry

**DOI:** 10.3389/fpsyt.2025.1630512

**Published:** 2025-08-19

**Authors:** Camille Jantzi, Valerie Moulin

**Affiliations:** ^1^ University Center of Legal Medicine (CURML), Geneva University Hospital, Geneva, Switzerland; ^2^ Université Grenoble Alpes, Laboratoire Inter-universitaire de Psychologie, Personnalité, Cognition et Changement Social-LIP/PC2S, Grenoble, France

**Keywords:** criminology, forensic psychiatry, ICD 11, personality disorder, psychiatric evaluation

## Abstract

This paper examines the implications of the transition from ICD-10 to ICD-11 for the diagnosis of personality disorders in forensic psychiatric evaluations. The ICD-11 introduces a dimensional approach, replacing the previous categorical system with a focus on severity and maladaptive personality traits. This shift addresses longstanding criticisms of the ICD-10, such as underdiagnosis, diagnostic instability, and lack of scientific validity. The new model classifies personality disorders by severity (mild, moderate, severe) and five trait domains, enhancing clinical nuance but also introducing challenges in continuity and communication. While the ICD-11 aims to improve diagnostic accuracy and treatment planning, concerns remain regarding overdiagnosis, increased stigma-especially among adolescents and the adequacy of trait coverage. The absence of validated diagnostic tools and clear severity thresholds further complicates forensic application. Preliminary studies suggest a dramatic increase in diagnosed cases under ICD-11, raising questions about the risk of pathologizing normative behavior and the potential for excessive intervention. The paper highlights the need for further research and careful implementation to balance improved recognition of personality pathology with the avoidance of unintended negative consequences in forensic practice.

## Introduction

Pre-sentencing forensic psychiatric evaluations represent a challenge in retrospective diagnosis. In these cases, determining whether the assessed individual was suffering from a “serious mental disorder” at the time of the act is paramount. (1, 2). This determination, as outlined by Article 19 of the Swiss Penal Code, assesses whether the disorder could have impaired their ability to appreciate the unlawfulness of their actions or act accordingly.

This issue is central to pre-sentencing evaluations, as stipulated by Article 20 of the Swiss Penal Code. The diagnosis influences both the type of therapeutic measures proposed and, to some extent, the evaluation of the individual’s dangerousness. It is essential to adopt an internationally recognized diagnostic framework to ensure consistency and reproducibility in medico-legal contexts (3, 4).

At the Forensic Psychiatry Unit in Geneva, the ICD-10 (International Classification of Diseases) was historically used. However, since January 1, 2022, the new ICD-11 classification has come into effect, necessitating training and adaptation. While some diagnostic areas remain unchanged, such as psychoses, others—like personality disorders, paraphilias, substance use disorders, and trauma-related disorders—have undergone major paradigm shifts (5, 6).

This paper focus on personality disorders, as this domain presents the most radical changes and clear implications for forensic psychiatric practice, specifically, the ICD-11 transitions from a categorical to a dimensional approach. Unlike the ICD-10, which described personality disorders using ten specific categories with detailed criteria, the ICD-11 prioritizes evaluating the severity of functional impairment and identifying predominant personality traits. This focus on severity is particularly relevant in forensic settings, as it is a major predictor of outcomes (7). In forensic populations, the prevalence of personality disorder is high (8, 9) and could impact the evaluation of the needs for intervention to reduce recidivism (10).

The aim of this paper is to highlight the changes and their impact on forensic psychiatric evaluations.

## Context and definitions

### The need for change

Numerous authors have highlighted the challenges associated with the ICD-10 categorical classification of personality disorders (7, 11, 12), advocating for a paradigm shift. A significant critique of the ICD-10 was its overly strict diagnostic criteria, which often led to underdiagnosis. The suspicion of underdiagnosis arose from the fact that the prevalence of personality disorders, regardless of type, was consistently reported to be much lower than other mental health disorders in research databases (13, 14).

Additionally, among those diagnosed with personality disorders, 95% were classified under only three categories: “antisocial personality,” “emotionally unstable personality,” or “mixed personality disorder” (15, 16). This suggested that existing categories did not adequately capture the clinical presentations observed by practitioners. Another criticism was the instability of diagnoses over time, despite requiring symptom stability over time as a diagnostic criterion.

The primary critique, however, was the lack of scientific validity behind these categories, which were based solely on clinical observations and historical work on personality disorders. A meta-analysis concluded that personality disorders represent extreme values along a continuum between normal and pathological personality traits, as described in the Five-Factor Model (17). This model, derived from Cattell’s personality theory (18), was initially considered during the development of the DSM-5 but ultimately abandoned in favor of a categorical approach (19).

The dimensional model views personality disorders as existing on a continuum, ranging from normal personality traits to maladaptive extremes. This allows for a more nuanced understanding of personality pathology, rather than rigidly categorizing individuals as having or not having a disorder. Dimensional models reduce issues like diagnostic overlap and the “not otherwise specified” (NOS) category seen in categorical systems. They provide incremental validity by describing all personality traits and eliminating artificial boundaries between disorders. Dimensional frameworks account for the fluctuating expression of traits depending on environmental factors or stress levels, making them more adaptable to changes in an individual’s condition over time (20).

## ICD-11 classification of personality disorders

### General diagnostic features

Personality disorders are defined by impairments in self-functioning (e.g., identity, self-worth, self-direction) and/or interpersonal functioning (e.g., ability to maintain relationships, empathy, conflict management). These impairments must be pervasive, persistent, and not attributable to developmental stages or cultural norms.

### Severity levels

Personality disorders are classified based on severity: mild, moderate, or severe. Severity reflects the degree of dysfunction in self and interpersonal functioning and the associated risks (e.g., harm to self or others).

### Trait domains

Five maladaptive personality trait domains describe the nature of personality dysfunction:

- **Negative Affectivity**: Emotional instability, anxiety, and depressive tendencies.
- **Detachment**: Social withdrawal, avoidance of intimacy.
- **Dissociality**: Disregard for others, manipulativeness.
- **Disinhibition**: Impulsivity, irresponsibility.
- **Anankastia**: Perfectionism, rigidity.

### Borderline pattern qualifier

A specific “borderline pattern” qualifier can be added if the individual meets criteria similar to DSM-5’s Borderline Personality Disorder (e.g., emotional instability, fear of abandonment).

## Other relevant diagnostic entities

### Schizotypal personality disorder

In the ICD-11, schizotypal personality disorder (6A22) is classified under psychotic disorders and retains the same criteria as in the ICD-10. This alignment emphasizes the close relationship between schizotypal traits and psychotic disorders, maintaining consistency in its diagnostic approach.

### Dissocial or disruptive behavior disorders

A key distinction in the ICD-11 is the removal of age-specific classifications for many disorders, allowing diagnoses to be applied across all ages unless explicitly restricted (e.g., reactive attachment disorder, which can only be diagnosed before the age of five). For instance, the Oppositional Defiant Disorder (6C90) and the Conduct Disorder (6C91), previously restricted to children and adolescents, can now theoretically be diagnosed in adults.

This diagnosis may apply to adults, particularly young adults with an adolescent-like context, though caution must be exercised to avoid the over-psychiatrization of criminal behavior.

### Intermittent explosive disorder

Intermittent Explosive Disorder (6C73), part of impulse-control disorders, is highly relevant in forensic settings due to its implications for evaluating legal responsibility. The disorder is characterized by Brief episodes of verbal or physical aggression or property destruction reflecting an inability to control aggressive impulses.

This disorder is particularly pertinent for individuals who commit repeated acts of violence without meeting the criteria for a personality disorder. Its diagnosis could significantly influence assessments of volitional capacities and legal accountability.

### Complex post-traumatic stress disorder

The ICD-11 introduces Complex Post-Traumatic Stress Disorder (CPTSD, 6B41), defined as a disorder that may develop following exposure to events of an extremely threatening or horrifying nature. In addition to meeting the criteria for PTSD, CPTSD is characterized by severe and persistent difficulties in affect regulation, self-perception, and interpersonal relationships.

This diagnosis aims to differentiate CPTSD from personality disorders, particularly emotionally unstable personality disorder, ensuring appropriate therapeutic interventions. Advocacy groups, such as #TraumaNotPD, have campaigned for this distinction to prevent misdiagnosis and mistreatment (21).

### Personality difficulty

The ICD-11 also includes the non-diagnostic category Personality Difficulty (QE50.7), which corresponds to the former “accentuated personality traits” category. This designation is intended for individuals who exhibit specific personality traits that may complicate treatment but do not meet the criteria for a personality disorder.

In forensic settings, using this category requires caution to avoid pathologizing normal psychological defenses under stress. Additionally, it should not be confused with a mental disorder that might affect legal responsibility.

## Literature review

### Increased incidence

The ICD-11 has sparked considerable academic discussion, even prior to its official release. Many authors have advocated for this new classification, particularly for its redefinition of personality disorder diagnosis (7, 11, 12). The aim of the new classification was to increase the recognition and diagnosis of personality disorders, and studies indicate that this goal has been achieved (22). This outcome is primarily due to the broader diagnostic criteria, including a shorter temporal requirement (only two years for diagnosis) and the allowance for diagnosing minors.

A meta-analysis highlighted the clinical utility of the new classification but also identified limitations, particularly regarding its clarity and ease of communication with patients and their families (23).

### Diagnostic tools

Currently, limited clinical studies validate the ICD-11 classification, largely due to a lack of established diagnostic tools. Several scales are under exploration, such as the Personality Inventory for ICD-11 (PiCD), a 60-item self-assessment scale evaluating the five trait domains, which also has an informant-report form (PiCD-IRF) (24–26). Another tool, the Standardized Assessment of Severity of Personality Disorder (SASPD), is a 9-item self-report questionnaire assessing functional impairment of personality traits (27). A third tool, the Personality Disorder Severity Scale (PDS-ICD-11), evaluates severity using 14 items and identifies severe personality disorders with a threshold score of 175, though it struggles to differentiate between mild and moderate disorders (28). The DSM-5 Personality Disorder Instrument (PDI), developed as an experimental tool for assessing dimensional personality pathology, has also been evaluated in forensic settings. Preliminary studies indicate that the PDI may offer valuable insights into the assessment of personality dysfunction in offenders, providing incremental validity over categorical approaches (29). However, its routine use in forensic practice remains limited, and further research is needed to establish its utility and comparability with ICD-11-based assessments.

In forensic settings, the assessment of psychopathy may also be useful but must be used with care regarding the stigma around this denomination. The gold standard for the assessment of psychopathy in adults is the Psychopathy Checklist-Revised (PCL-R; 30), a 20-item clinician-rated instrument that evaluates interpersonal, affective, and behavioral features associated with psychopathy. The PCL-R has been extensively validated in forensic and clinical populations and is widely used for risk assessment and research purposes (30, 31). For young people, two key instruments are widely used: the Psychopathy Checklist: Youth Version (PCL-YV; 32), which is adapted for adolescents, and the Comprehensive Assessment of Psychopathic Personality (CAPP; 33). The PCL-YV is considered the gold standard for assessing psychopathic traits in youth, while the CAPP provides a theoretically grounded, trait-based approach compatible with dimensional models such as ICD-11. However, neither instrument has been specifically validated for use within the ICD-11 personality disorder framework, and their integration into new diagnostic paradigms remains an open question.

For borderline traits specifically, the Borderline Pattern Scale (BPS) is being tested (34). Notably, studies in forensic settings have shown poor concordance between self-reports and informant reports, underscoring the importance of avoiding self-assessment tools in forensic evaluations (35).

### Five-trait model

Validation studies of the five-trait model suggest that it may conceptually be more accurate as a four-trait model. This adjustment merges anankastia and disinhibition, as they represent opposite poles of the same dimension. This alignment reflects the challenges of diagnosing individuals who exhibit traits of both impulsivity and emotional repression simultaneously (36–39).

### Genetic and neuroimaging research

Recent advances in genetic and brain imaging research have not yet provided robust biomarkers or clear neurobiological correlates for the ICD-11 personality disorder revisions. While some studies have explored heritability and neural correlates of personality pathology, findings remain inconsistent and do not currently support the use of genetic or neuroimaging data for diagnosis or for distinguishing between ICD-10 and ICD-11 models (40, 41). This highlights a significant limitation in the current scientific foundation for the ICD-11 revisions, emphasizing the need for further interdisciplinary research.

### Severity and prognosis

The evaluation of disorder severity has been shown to inform treatment intensity and prognosis (42, 43). While there is limited forensic literature directly examining the predictive validity of ICD-11 personality disorder severity for recidivism risk, recent research has extensively explored the relationship between adolescent psychopathy and long-term offending. For example, McCuish et al. (44) conducted a large cohort study following youth with psychopathic traits into adulthood, demonstrating significant predictive validity for recidivism. These findings suggest that certain personality pathology dimensions, particularly those related to psychopathy, may have important implications for risk assessment, even if direct evidence for ICD-11 categories is still emerging.

### Critiques

Despite its advantages, the ICD-11 has faced several criticisms. One justification for the shift to a dimensional model was to reduce the stigma associated with personality disorder diagnoses. However, some researchers question whether this goal will be achieved. In fact, they argue that the broader diagnostic criteria and increased prevalence might amplify stigma, especially when diagnosing adolescents. For example, labeling adolescents with personality disorders could lead to self-fulfilling prophecies, negatively impacting their self-image and increasing marginalization (45, 46).

A study by Perkins et al. (47) found that being labeled as having a personality disorder can harm an individual’s self-esteem and does not necessarily improve access to care. Moreover, clinicians may perceive patients with personality disorders more negatively, often overlooking other symptoms like anxiety or depression, interpreting them solely through the lens of the personality disorder (48, 49).

The “Borderline Pattern” descriptor in the ICD-11 has also been criticized for lacking scientific validity, as it is rarely addressed in clinical studies and relies solely on the five-trait model (50). Furthermore, certain traits, such as “Negative Affectivity,” are criticized for being overly broad and potentially applicable to a wide range of disorders (51). Lastly, critics argue that key personality types, such as avoidant, dependent, or narcissistic personalities, are inadequately captured in the ICD-11 framework (52).

### A problem of continuity

The introduction of the ICD-11 marks a clear break from previous diagnostic approaches, creating challenges in research and clinical practice. Conducting a literature review now requires attention to both ICD-10 and ICD-11 terminology to ensure comprehensive results. In forensic practice, continuity issues also arise when comparing past and present evaluations. Experts must navigate these differences by focusing on symptom descriptions rather than rigid diagnostic categories.

The lack of clearly defined thresholds for severity levels (e.g., mild, moderate, severe) in the ICD-11 could also lead to variability between examiners, potentially complicating reevaluations and forensic decisions. Preliminary studies suggest that the three-tiered severity model may lack empirical support and require further validation (28, 52).

Another important limitation in the current ICD-11 literature is the insufficient discussion of comorbidity, particularly with key neurodevelopmental disorders such as Autism Spectrum Disorder (ASD) and Fetal Alcohol Spectrum Disorder (FASD). Both ASD and FASD can present with overlapping features of personality dysfunction, complicating differential diagnosis and risk assessment in forensic populations (53, 54). The ICD-11 does not provide specific guidance on addressing these complex comorbidities, which remains a significant challenge for clinicians.

### In the forensic domain

The implications of the ICD-11 for forensic psychiatry remain insufficiently explored. Some researchers, such as Frances and Nardo (55), argue that the DSM-5 may be more suitable for forensic settings due to its perceived precision and reliability. They caution that the broader diagnostic criteria in the ICD-11 could lead to the pathologization of clinical presentations previously deemed borderline normal. Overdiagnosis in this context could result in excessive therapeutic recommendations for individuals who may not genuinely require intervention. The Risk-Need-Responsivity (RNR) model (56), now widely adopted in recidivism prevention frameworks, warns that excessively intensive interventions for low-risk offenders may paradoxically increase their risk of recidivism by fostering marginalization and stigma.

An English study conducted in a high-security forensic hospital (57) reported a prevalence of personality disorders of 30.8% using ICD-10 criteria, which rose dramatically to 100% when applying ICD-11 criteria. While the study’s authors emphasized the improved clinical validity of the ICD-11, the significant increase raises concerns about overly inclusive diagnostic thresholds. Notably, the ICD-11 diagnoses in this study were made retrospectively from medical records, rather than based on live assessments by clinicians. This methodological limitation might have amplified the apparent prevalence under the new classification system.

The study further identified predominant traits among forensic patients diagnosed with “antisocial personality disorder” and “emotionally unstable personality disorder.” For individuals with antisocial traits, dissociality and disinhibition were frequently observed, accompanied by low levels of negative affectivity. In contrast, patients classified under emotionally unstable personality disorder exhibited strong negative affectivity in addition to dissociality and disinhibition, often with the “borderline pattern” descriptor.

Efforts to map ICD-10 categories to ICD-11 traits have been made (52), such as linking “negative affectivity and detachment” to avoidant personalities, or “dissociality and anankastia” to narcissistic personalities. However, these attempts face limitations due to the fundamentally different paradigms of the two classification systems. The ICD-11’s emphasis on individualization resists rigid categorization, aiming instead to provide a flexible framework that describes psychological traits without confining individuals to predefined diagnostic “boxes.”

From a forensic perspective, the ICD-11’s dimensional model offers significant advantages. For instance, determining the severity of a personality disorder is critical when assessing legal responsibility (58). In Switzerland, the Penal Code requires forensic experts to evaluate whether an individual suffers from a “serious mental disorder” that significantly deviates from societal norms (Federal Court Ruling 116 IV 273). Mild personality disorders, especially under the broad ICD-11 criteria, are unlikely to meet this threshold.

The dimensional approach also addresses disparities in how responsibility mitigation was applied under the categorical model. Previously, individuals with narcissistic, antisocial, or obsessive-compulsive personality disorders often received less consideration for responsibility reduction. The ICD-11 encourages forensic experts to focus on clinically relevant traits, such as empathy deficits, impulsivity, and emotional regulation difficulties, rather than diagnostic labels. This nuanced analysis enhances the credibility of expert testimony in judicial settings.

Finally, the ICD-11 improves therapeutic recommendations by targeting specific maladaptive traits rather than attempting to resolve the entire disorder. This approach allows clinicians to set realistic goals, such as reducing impulsivity or improving emotional regulation, while acknowledging that complete remission may not be feasible. The dimensional framework also facilitates tracking improvements over time, as severity reductions can signal meaningful progress even if the diagnosis persists.

## Conclusion

The new ICD-11 classification introduces an important paradigm shift in the diagnosis of personality disorders. In forensic psychiatry, evaluating the severity of the disorder is essential, as jurisprudence requires determining if a defendant’s mental state significantly deviates from that of individuals who have committed similar acts. Furthermore, avoiding terms with stigmatizing connotations, such as “paranoid” or “narcissistic,” helps focus on the fundamental nature of the disorder as a mental health issue, for which the individual is not at fault.

The ICD-11’s broader and more flexible terminology encourages forensic experts to provide detailed descriptions of observed symptoms rather than relying solely on diagnostic criteria. This descriptive approach helps humanize the evaluated individual and facilitates a better understanding of their psychological functioning by judges and legal professionals.

However, the ICD-11 also presents challenges. The discontinuity with the ICD-10 complicates comparative evaluations, and many clinicians may continue using the previous categories due to their familiarity and ease of communication. Adopting the ICD-11 will require comprehensive training and widespread practice to ensure consistency in its application.

Further research is needed to validate diagnostic tools specific to the ICD-11 and to confirm the utility of the new classification in forensic contexts, such as predicting recidivism risk and guiding treatment recommendations. Despite these challenges, the dimensional approach offers a promising path toward more individualized, scientifically grounded, and less stigmatizing forensic psychiatric evaluations.
